# Sudden unexpected deaths in children: distinct entities

**DOI:** 10.1007/s00431-026-07328-0

**Published:** 2026-08-21

**Authors:** Thomas Hegyi, Barbara M. Ostfeld

**Affiliations:** The Division of Neonatology, Department of Pediatrics, and the SIDS Center of New Jersey, Robert Wood Johnson Medical School, Rutgers University, 1 Robert Wood Johnson Place, MEB 388B, New Brunswick, NJ 08901 USA

**Keywords:** SUDC, SUID, SUPC

## Abstract

Sudden unexpected deaths in early life have historically been grouped under broad, inconsistent terminology, limiting epidemiologic precision and mechanistic understanding. Standardized investigation and surveillance now permit clearer differentiation among distinct entities. The goal of this project is to synthesize epidemiologic, forensic, and molecular evidence supporting classification of sudden unexpected postnatal collapse (SUPC), sudden unexpected infant death (SUID), and sudden unexpected death in childhood (SUDC) as distinct entities, and to examine whether shared biologic vulnerability underlies cases across age groups. A narrative review used population-based epidemiologic studies, national and international surveillance data, forensic pathology reports, and molecular autopsy investigations, emphasizing standardized definitions, age-stratified analyses, and comprehensive death-scene investigation. SUPC is rare, occurs almost exclusively within the first postnatal week, and is linked to early caregiving context during physiologic transition. SUID is more common (approximately 101 per 100,000 US live births in 2022), peaks at 1 to 4 months, and shows robust associations with modifiable sleep-environment factors. SUDC is rare, occurs predominantly after age 1, and lacks consistent environmental risk factors; data suggest contributions from autonomic instability or seizure susceptibility. Molecular autopsy studies in SUID and SUDC have identified variants in genes governing cardiac conduction, autonomic regulation, and neuronal excitability, suggesting shared intrinsic vulnerability in a subset, though cohorts remain small. *Conclusions*: SUPC, SUID, and SUDC are distinct entities warranting separate classification and prevention strategies. Emerging but limited molecular data support further investigation of cross-age biologic vulnerability. Accurate classification, standardized investigation, and molecular integration are needed to advance the field.

**Supplementary Information:**

The online version contains supplementary material available at https://doi.org/10.1007/s00431-026-07328-0.

**Table:** 

**What is Known:** • *Sudden unexpected deaths in infancy and early childhood (SUPC, SUID, SUDC) have traditionally been groupedunder broad, inconsistently applied terminology, which has limited epidemiologic precision and mechanisticunderstanding of these events*. • *Each entity shows a distinct age distribution and risk profi le: SUPC occurs almost exclusively in the fi rst postnatalweek and relates to early caregiving context, SUID peaks at 1–4 months and is strongly tied to modifi able sleep-environment factors, and SUDC occurs predominantly after age 1 without a consistent environmental risk pattern*.
**What is New:** • *This review synthesizes standardized, age-stratifi ed epidemiologic and forensic data to support classifying SUPC,SUID, and SUDC as distinct entities requiring separate prevention strategies, rather than treating them as a singlecontinuum*. • *Molecular autopsy fi ndings across SUID and SUDC have identifi ed variants in genes governing cardiac conduction,autonomic regulation, and neuronal excitability, raising the novel possibility of a shared intrinsic biologicvulnerability that cuts across age groups despite their distinct epidemiologic profi les*.

## Introduction

Sudden unexpected deaths in early life encompass a heterogeneous group of clinical and forensic phenomena that have historically been described using overlapping and inconsistently applied terminology. This lack of precision has persisted despite advances in death-scene investigation and cause-of-death classification, resulting in continued aggregation of fundamentally distinct entities within surveillance systems and the literature [1, 2].

The entities sudden unexpected postnatal collapse (SUPC), sudden unexpected infant death (SUID), and sudden unexpected death in childhood (SUDC) can now be distinguished by reproducible epidemiologic and contextual features, and they are increasingly used as discrete categories within national and international epidemiologic surveillance and forensic investigation frameworks [3, 4]. Although each involves the sudden death of an apparently healthy infant or child, they differ substantially in age distribution, environmental context, associated risk factors, and prevailing mechanistic hypotheses.

Failure to distinguish among these entities has important consequences. Aggregation obscures true incidence patterns, confounds identification of risk factors, and encourages inappropriate extrapolation of prevention strategies across age groups [1, 2]. Interventions developed for sleep-related infant death, for example, cannot be assumed to apply to neonatal collapse during the delivery hospitalization or to unexplained deaths in toddlers [5, 6].

At the same time, emerging evidence from molecular autopsy investigation [7, 8] and from neurobiological studies of autonomic and respiratory control [9] suggests that intrinsic vulnerabilities, particularly those affecting autonomic regulation, cardiac conduction, and neuronal excitability, may contribute to a subset of unexplained deaths across infancy and childhood. The triple-risk model, originally proposed to explain sudden infant death, posits an interaction between a vulnerable individual, a critical developmental period, and an external stressor [10]. It remains uncertain whether this vulnerability is confined to infancy or persists in some individuals beyond the first year of life.

This review synthesizes current evidence supporting the classification of SUPC, SUID, and SUDC as distinct entities and examines the extent to which shared biologic substrates may underlie a subset of unexplained deaths across the pediatric age spectrum.

## Methods

A narrative review was performed, drawing on epidemiologic studies, national and international surveillance datasets, forensic pathology literature, and consensus statements [1–4, 11]. Studies were prioritized if they employed standardized case definitions, age-stratified analyses, and comprehensive death-scene investigation protocols. Molecular autopsy and genetic studies were included to evaluate emerging evidence of intrinsic biologic susceptibility [7, 8]. Findings were synthesized qualitatively to compare incidence, risk factors, clinical context, and investigative outcomes across SUPC, SUID, and SUDC.

## Results

### Epidemiology

SUPC is rare and confined to the immediate postnatal period. In a systematic review of 398 published cases, reported incidence varied widely by case-ascertainment method and definition, ranging from approximately 2.6 to 133 per 100,000 live births, Lower figures are typical of passive national surveillance and higher figures of active, single-center surveillance [5]. When a defined time frame was reported, events clustered in roughly equal thirds within the first 2 h after birth, within the first 24 h, and within the remainder of the first postnatal week [5]. Outcomes are poor: published cohorts report death or severe neurologic sequelae in roughly half of cases, with the remainder showing variable recovery [5].

SUID is substantially more common. National surveillance data report approximately 3700 SUID deaths in the USA in 2022, a rate of approximately 101 per 100,000 live births [3, 4, 12]. This is higher than the ≈3500 annual deaths commonly cited in national clinical guidance, a figure derived from earlier-period averages; the most recent single-year data indicate that the SUID rate has not continued to decline since 2020 and, if anything, has risen modestly [3, 4, 6, 12] Deaths peak between 1 and 4 months of age. Within this category, causes include sudden infant death syndrome (SIDS), accidental suffocation and strangulation in bed, and deaths classified as unknown cause after investigation [3].

### International and European comparisons

US SUID rates are substantially higher than rates reported for Western Europe, where the analogous entity is generally termed sudden unexpected death in infancy (SUDI) [13]. A 14-country analysis of Western European national statistics for 2005–2015 reported an overall SUDI rate of 34.9 per 100,000 live births, declining from 40.2 to 29.9 per 100,000 over the study period, roughly one-third the contemporaneous US rate [14]. Part of this difference reflects genuine variation in exposure to modifiable risk factors, but part reflects documented differences in death-scene investigation intensity and cause-of-death coding practice. An eight-country comparison found that the proportion of SUDI deaths coded as SIDS ranged from 32.6% in Japan to 72.5% in Germany, and that overall SUDI rates ranged more than fivefold, from 0.19 per 1000 live births in the Netherlands to 1.00 per 1000 in New Zealand, using the same standardized ICD-10 code set [15]. These findings indicate that a meaningful portion of the cross-national gap in reported rates reflects inclusion and certification practice, specifically, how liberally national systems apply the SIDS/R95 code versus ill-defined or undetermined codes, rather than true differences in underlying risk alone [15]. For this reason, we use SUID (the US/CDC terminology) throughout this review, while noting that SUDI is the operative term, with a distinct grading system based on investigation completeness, in much of the European and UK literature [13].

SUDC is considerably rarer, with an estimated incidence of 0.1 to 1.4 per 100,000 children aged 1 to 4 years [16, 17]. These deaths occur predominantly during sleep, often at home, and show seasonal clustering (winter) and a modest male predominance [17].

### Risk factors and clinical context

SUPC is associated with situational factors in the early postnatal period, including prone positioning during skin-to-skin contact, unsupervised breastfeeding, swaddling, primiparity, and caregiver fatigue. Events occur during a period of physiologic transition when respiratory and autonomic control remain immature [5].

SUID demonstrates strong and reproducible associations with modifiable sleep-environment factors. Prone sleep position, soft bedding, bedsharing, maternal smoking, and prenatal substance exposure have been consistently identified as major risk factors and form the basis of the American Academy of Pediatrics’ 2022 updated safe-sleep recommendations [6]. Public-health campaigns targeting these factors have been associated with substantial reductions in mortality in the USA and internationally. Supine-positioning campaigns have been linked to 50–90% reductions in SIDS rates in multiple countries, reinforcing their causal relevance, although rate reductions have plateaued since 2000 and disparities persist [6, 9, 14].

SUDC lacks consistent environmental risk factors of comparable strength. Registry-based studies have reported a substantially higher prevalence of febrile seizures among SUDC decedents than among matched controls, and a tendency for children to be found prone during sleep, but these findings do not establish a clear environmental mechanism [18]. The absence of reproducible external risk factors suggests that intrinsic biologic processes may play a more prominent role, a hypothesis reinforced by video-recorded terminal events in a subset of cases showing brief convulsive activity immediately preceding death [19].

### Pathology and emerging biologic insights (Table 1)

**Table 1:** Comparative clinical, epidemiologic, investigative, and molecular features of SUPC, SUID, and SUDC

Feature	SUPC	SUID	SUDC	Key refs
Age range	Birth to ≤ 7 days (predominantly first 72 h)	Birth to < 1 y (peak 1–4 mo)	> 1 y (peak 1–4 y)	5, 16, 17
Incidence	≈2.6–133 per 100,000 live births (varies by case ascertainment)	≈101 per 100,000 live births (≈3700 deaths/y, USA, 2022)	≈0.1–1.4 per 100,000 children aged 1–4 y	3, 4, 5, 16, 17
Temporal pattern	Immediate postnatal transition; hospital postpartum unit; often daytime	Peak 1–4 mo; home sleep environment; nocturnal predominance	During sleep, often at home; seasonal (winter) clustering; modest male excess	5, 6, 17
Standardized case definition	Emerging consensus: gestational age ≥ 34 wk, collapse ≤ 7 d	Umbrella ICD-10–based category (SIDS, ASSB, unknown cause)	San Diego definitional approach for SIDS informs SUDC criteria; UK/EU SUDI grading (Ia–III) differs from US usage	5, 13, 21
Primary risk structure	Situational: prone positioning during skin-to-skin contact, unsupervised breastfeeding, swaddling, primiparity, caregiver fatigue	Modifiable sleep-environment factors: prone sleep, soft bedding, bedsharing, maternal smoking, prenatal substance exposure	Febrile seizures over-represented; possible prone position; no consistent environmental hazard identified	5, 6, 18
Role of environment	Immediate and proximal to caregiving context	Dominant and modifiable; target of national prevention campaigns	Limited or inconsistent	5, 6
Autopsy findings	Often nonspecific; survivors show hypoxic-ischemic injury on neuroimaging	Variable; SIDS remains a diagnosis of exclusion; asphyxial findings in some cases	Typically, negative or subtle; increasing focus on hippocampal/temporal-lobe findings	5, 9, 11, 20
Dominant mechanistic hypothesis	Acute airway compromise or impaired arousal during transition	Triple-risk model: vulnerable infant + critical developmental period + exogenous stressor	Possible autonomic instability or seizure-related mechanism; overlap with SUDEP proposed	6, 9, 10, 18
Genetic testing	Rarely performed; sparse data	Increasingly incorporated; variants in cardiac-conduction and autonomic-regulation genes	Strongly recommended in unexplained cases; de novo variants disrupting Ca^2^⁺-regulatory and cardiac/seizure-gene networks	7, 8
Prevention strategy	Supervised skin-to-skin care; safe positioning; early postpartum monitoring	AAP safe-sleep recommendations; national campaigns (USA and internationally)	No established population-level strategy; research- and registry-focused	5, 6, 14, 15
Data systems and registries	Limited centralized tracking	National SUID Case Registry and Child Death Review	SUDC Registry and Research Collaborative; developing international consortia	2, 3, 16
Limitation of the investigation	Event rarely witnessed; context-specific	Persistent inter-jurisdictional variability in cause-of-death classification despite protocols	Negative autopsy and absence of an environmental explanation; small cohort sizes	1, 2, 11, 15

*ASSB*, accidental suffocation and strangulation in bed; *SIDS*, sudden infant death syndrome; *SUDEP*, sudden unexpected death in epilepsy; *SUDI*, sudden unexpected death in infancy (European/UK terminology)

In SUPC, autopsy findings are often nonspecific, and in survivors, neuroimaging typically demonstrates hypoxic-ischemic injury consistent with acute cardiorespiratory compromise. The timing and context of these events support a primary role for transient airway obstruction or impaired arousal during early physiologic adaptation [5].

In SUID, a comprehensive investigation may identify asphyxial mechanisms in some cases, whereas SIDS remains a diagnosis of exclusion after negative autopsy, scene investigation, and clinical-history review, a determination subject to well-documented inter-jurisdictional variability [9, 11].

In SUDC, autopsies are frequently unrevealing. Neuropathologic studies have described an association with hippocampal and temporal-lobe developmental abnormalities in a subset of toddlers who died suddenly with a febrile-seizure history, and increasing attention has focused on this finding alongside genetic susceptibility [17, 20]. Molecular autopsy studies in SUID and SUDC have identified pathogenic and likely pathogenic variants in genes associated with cardiac channelopathies, autonomic dysfunction, and epilepsy syndromes. In a trio whole-exome sequencing study of 124 SUDC decedents and their parents, de novo mutations were significantly enriched in genes regulating intracellular calcium signaling shared between cardiomyocytes and neurons [7, 8]. Estimated diagnostic yields for pathogenic or likely pathogenic variants in these cohorts are in the range of roughly 5–15%, though estimates vary by cohort, sequencing platform, and variant-classification criteria [7, 8]. Although the clinical significance of many variants remains uncertain, the recurrence of findings across cohorts suggests that a subset of unexplained deaths may share underlying biologic vulnerability. This hypothesis, given the modest size of the cohorts studied to date, should be regarded as provisional pending replication in larger, independent samples [7, 8].

## Discussion

The evidence supports the conclusion that SUPC, SUID, and SUDC are distinct clinical entities rather than variations along a single continuum. Each is characterized by a unique age distribution, environmental context, and pattern of associated risk factors, and recognition of these distinctions is essential for accurate surveillance, valid epidemiologic inference, and the development of effective prevention strategies [1, 2].

SUPC is best understood as a disorder of early postnatal transition, occurring in a narrowly defined temporal window and closely linked to immediate caregiving conditions [5]. SUID, in contrast, reflects the interaction between infant vulnerability and a modifiable sleep environment during a critical developmental period, consistent with the triple-risk model [6, 10]. SUDC occupies a distinct domain, characterized by rarity, the absence of consistent environmental triggers, and growing evidence pointing to neurologic or electrophysiologic mechanisms that may overlap with sudden unexpected death in epilepsy [8, 18].

### Other attempts at classification

The tripartite framework used here is not the only classification system in use, and it is worth situating it against alternatives. In the USA, the “San Diego” definitional and diagnostic approach to SIDS established graded categories (IA, IB, II) based on the completeness of death-scene investigation and the presence of minor risk factors, an approach that improved diagnostic consistency but was developed specifically for infant deaths and does not extend naturally to childhood [21]. In the UK and much of Europe, the SUDI classification proposed by Blair, Byard, and Fleming instead grades cases from Ia to III according to the thoroughness of investigation, deliberately avoiding the term “undetermined” [13]. Most recently, the 3rd International Congress on Sudden Infant and Child Death explicitly called for harmonized, internationally comparable classification criteria spanning infancy and childhood, concluding that persistent inconsistency in cause-of-death determination, not merely terminology, continues to hinder surveillance, prevention, and research [2]. The framework adopted in this review is broadly consistent with that recommendation but is offered as a synthesis rather than a competing nomenclature.

A related tension deserves direct comment. This review argues against aggregating SUPC, SUID, and SUDC, yet it also uses SUID itself as an umbrella term spanning SIDS, accidental suffocation and strangulation in bed, and deaths of unknown cause [3]. We do not regard this as contradictory, but the distinction is worth making explicit. SUID aggregates causes of death that share a common age window, a common environmental risk profile, and a common prevention strategy, and combining them is defensible, indeed necessary, for surveillance and public-health purposes, since case-by-case discrimination among these subcategories is itself subject to substantial and well-documented inter-jurisdictional variability [11]. Aggregating across SUPC, SUID, and SUDC, by contrast, combines entities that differ in age, environmental context, and risk-factor structure, and is not defensible for the same purposes. The principle we advance is not that aggregation is always wrong, but that it should track shared mechanism and shared prevention target rather than superficial phenomenological similarity (“sudden, unexpected, and unexplained”).

### Shared biologic vulnerability: a provisional hypothesis

The possibility of shared biologic susceptibility across age groups warrants careful, and appropriately cautious, consideration. The triple-risk framework provides a useful starting point, but its traditional restriction to infancy may be overly narrow [10]. Molecular autopsy data suggest that variants affecting autonomic regulation, cardiac excitability, or neuronal stability may persist beyond the first year of life in some individuals, with different age-specific stressors, ranging from sleep-related challenges in infancy to febrile illness in early childhood, potentially precipitating fatal events in a susceptible substrate [7, 8]. This hypothesis, however, rests on cohorts that remain small. The largest SUDC trio-sequencing study to date included 124 families, and comparable SUID molecular autopsy cohorts are of similar or smaller scale [7, 8]. Given that SUDC and SUPC are, by a wide margin, the rarer of the three entities discussed here, claims of a shared biologic substrate spanning all three should be read as hypothesis-generating rather than established. We regard this as a promising direction for future, adequately powered, multi-registry research rather than a settled conclusion.

### Persistent limitations in classification

The present synthesis also addresses a persistent limitation in the field, which is inconsistency in classification. Variability in cause-of-death assignment has been well documented even within standardized US systems, and is, if anything, more pronounced across national borders, as illustrated by the more than twofold cross-country range in the proportion of SUDI deaths coded as SIDS versus ill-defined causes [11, 15]. Without greater clarity, efforts to identify mechanisms, whether environmental, infectious, or genetic, remain fundamentally constrained [2].

Hypotheses emphasizing inflammation or infection as primary causes of sudden death have been considered extensively but have not achieved consistent empirical support across populations [9]. While such factors may act as modifiers or triggers in individual cases, they do not account for the majority of observed deaths and should be interpreted within a broader framework of vulnerability and exposure. Similarly, proposed associations between routine childhood immunization and SIDS have not been supported by the epidemiologic evidence. A meta-analysis of case–control studies found no increase in risk following immunization and, if anything, a modest inverse association, and this literature should not be taken to influence scientific or public-health discourse in this area [22].

Progress in understanding sudden unexpected death across early life will depend on maintaining a broad, multidisciplinary, and internationally coordinated approach. Advances in molecular autopsy, standardized death-scene investigation, and integrated, harmonized data systems provide an opportunity to move beyond descriptive classification toward mechanistic insight [2, 7, 8, 11]. Such progress, however, depends first on recognizing that the entities under study are not interchangeable, and second on acknowledging the current limits of the evidence for shared mechanism across them.

In summary, clear differentiation of SUPC, SUID, and SUDC is both justified by current evidence and necessary for further advancement. At the same time, the possibility of shared biologic vulnerability across a subset of cases introduces an important, but still provisional, direction for future investigation. These perspectives are complementary rather than contradictory and together provide a more coherent framework for research and prevention.

## Supplementary Information

Below is the link to the electronic supplementary material.

ESM 1 DOCX (165 KB)

## Data Availability

No datasets were generated or analysed during the current study.

## Abbreviations

SUPC: Sudden unexpected postnatal collapse
SUID: Sudden unexpected infant death
SUDC: Sudden unexpected death in childhood
