# Supplementary material for: Sudden unexpected deaths in children: distinct entities
Source: Eur J Pediatr. 2026 Aug 21;185(9):685. doi: 10.1007/s00431-026-07328-0 (PMC13498490; doi:10.1007/s00431-026-07328-0)
Supplement: Supplementary file 1 [file 431_2026_7328_MOESM1_ESM.docx]

April 30, 2026

European Journal of Pediatrics

Dear Editors,

Submission ID: c6a001bf-31c8-4a02-a3aa-edd8cddfa7b2

We write to resubmit the enclosed manuscript, "Sudden Unexpected Deaths in Children: Distinct Entities," for consideration as an article in the European Journal of Pediatrics.

Sudden infant death remains one of the leading causes of postnatal mortality in high-income countries, yet the terminology used in clinical practice and public health often conflates three epidemiologically distinct entities: sudden unexpected postnatal collapse (SUPC), sudden unexpected infant death (SUID), and sudden unexpected death in childhood (SUDC). That conflation has practical consequences. Prevention strategies effective for sleep-related SUID are not applicable to neonatal SUPC, and SUDC increasingly points toward neurologic and genetic substrates that neither safe-sleep messaging nor neonatal monitoring addresses. Clinicians who do not distinguish these entities risk applying the wrong conceptual framework to families who deserve the right one.

Our article argues for maintaining clear boundaries among these three syndromes while opening a distinct question: whether a subset of unexplained deaths across infancy and early childhood shares intrinsic biologic vulnerabilities that persist beyond the first year of life. Emerging molecular autopsy findings in SUDC, involving variants in cardiac channelopathy, epilepsy, and autonomic dysfunction genes, parallel findings in SUID and raise the possibility that the triple-risk model of SIDS may require extension to older children. We propose an extended-vulnerability framework and argue that expanding genetic investigation across the pediatric age spectrum is warranted, while explicitly stating what the current evidence does and does not support.

We believe this synthesis is timely. Molecular autopsy is becoming more widely available, SUDC registries are expanding, and clinicians increasingly face questions from families of children who die beyond infancy without explanation. An article that clarifies the distinctions, their clinical implications, and frames the emerging biological questions with appropriate precision would serve both the practicing clinician and the research community.

This manuscript has not been submitted elsewhere and is not under consideration at another journal. All authors have reviewed and approved the final version and have no conflicts of interest to declare.

We welcome the opportunity to provide a response to the comments provided by the previous evaluators of the manuscript. Since the reviews were extensive, they are not duplicated below.

Response to Reviewer 1

First, the assertion that this manuscript “states the obvious” fundamentally misrepresents both the purpose and the current state of the field. The persistent conflation of SUPC, SUID, and SUDC in surveillance systems, publications, and even policy discussions is well documented. This lack of nosologic clarity has practical consequences. It distorts epidemiologic interpretation, confounds mechanistic research, and leads to the misapplication of prevention strategies across biologically and clinically distinct conditions. Our manuscript does not merely restate known facts but systematically synthesizes disparate literatures to demonstrate that these entities are separable on epidemiologic, contextual, and mechanistic grounds, while also advancing a unifying hypothesis regarding shared biologic vulnerability. That dual argument is neither trivial nor widely articulated in a single framework.

Second, the reviewer’s claim that we treat SUID or SIDS as “purely” environmental is incorrect. The manuscript explicitly discusses intrinsic biologic vulnerabilities, including genetic variants affecting autonomic regulation, cardiac conduction, and neuronal excitability, and it situates these within an expanded vulnerability framework that extends beyond infancy. We explicitly reference and engage with the triple risk model, not as dogma, but as a platform for extension. The reviewer’s criticism therefore addresses a position we did not take.

Third, the reviewer’s emphasis on inflammation, infection, and vaccine exposure reflects a selective and, in parts, speculative interpretation of the literature rather than a balanced appraisal of current evidence. While inflammatory pathways and infectious triggers have been explored in SIDS research, they remain neither necessary nor sufficient explanations for the majority of cases and have not achieved the level of reproducible, causal inference required to anchor a contemporary synthesis. The inclusion of vaccines as a putative causal factor is particularly concerning, as it is not supported by robust epidemiologic evidence and risks introducing unsupported inference into a field where public health messaging must remain evidence based and precise.

Importantly, our review does not exclude the possibility that infection or inflammation may act as modifiers or triggers within a broader vulnerability framework. Rather, we intentionally avoid overextension beyond the strength of available data. A narrative review, particularly one intended to clarify classification and conceptual structure, must prioritize reproducibility, methodological rigor, and consensus-supported findings over isolated or controversial hypotheses.

Fourth, the reviewer’s suggestion that infection should be “the focus” of future research represents a narrowing of inquiry that is inconsistent with the multidisciplinary direction of the field. Advances in molecular autopsy, genomics, neurophysiology, and integrated death investigation argue strongly for a pluralistic research agenda. Our manuscript reflects that reality by emphasizing the need for standardized classification, rigorous investigation, and biologically informed approaches that can accommodate multiple pathways rather than privileging a single mechanism.

Finally, the critique does not engage with the central contribution of the manuscript which demonstrates that accurate classification is a prerequisite for meaningful research. Without clear separation of SUPC, SUID, and SUDC, any discussion of mechanism, whether environmental, inflammatory, genetic, or otherwise, remains inherently confounded. The reviewer’s argument, in effect, reinforces the very problem the manuscript seeks to correct.

For these reasons, we believe the reviewer’s comments are based on a misreading of the manuscript and on the introduction of unsupported or tangential hypotheses that fall outside the scope and intent of this work. We respectfully request that these criticisms not be used to guide editorial judgment and that we're allowed to correct items identified by the second reviewer and resubmit the manuscript.

Response to Reviewer 2

- We added the requested references as requested for the studies, surveillance data and consensus statements.
- The table has been corrected to clarify that the incidence noted applies to the USA
- The error referring to reference 6 has been corrected.

Sincerely,

Thomas Hegyi, MD

Thomas Hegyi, M.D.

Program Director, Division of Neonatology

Professor, Department of Pediatrics

Director, SIDS Center of New Jersey

Robert Wood Johnson Medical School

Rutgers, The State University of New Jersey
